# Curative outcomes with metal-containing TCM in diabetic foot ulcers unresponsive to standard therapy: a case series

**DOI:** 10.3389/fendo.2026.1736055

**Published:** 2026-05-08

**Authors:** Yifan Zhang, Cai Yu, Xiaohan Qu, Qiannan Li, Guangyu Dai, Wanying Long, Hui Kong, Huihua Qu, Xuying Xu, Yue Zhang, Yan Zhao

**Affiliations:** 1 School of Traditional Chinese Medicine, Beijing University of Chinese Medicine, Beijing, China; 2 Department of Carbuncle and Ulcers, Beijing Daxing District Hospital of Integrated Chinese and Western Medicine, Beijing, China; 3 Department of Outpatient, Beijing Daxing District Hospital of Integrated Chinese and Western Medicine, Beijing, China; 4 Center of Scientific Experiment, Beijing University of Chinese Medicine, Beijing, China; 5 Department of Ulcerative Vascular Surgery, Beijing Hospital of Traditional Chinese Medicine, Beijing, China; 6 School of Life Sciences, Beijing University of Chinese Medicine, Beijing, China

**Keywords:** case report, diabetic foot ulcers, Huafu Shengji ointment, regenerative repair, topical conservative therapy

## Abstract

**Background:**

Diabetic foot ulcers (DFUs) are among the most severe complications of diabetes, often resulting in prolonged healing, recurrent infections, and a high risk of amputation. Despite comprehensive standard treatments, many patients develop chronic non-healing ulcers due to poor tissue perfusion and immune dysregulation. Traditional Chinese medicine, such as Huafu Shengji ointment (HFSJO), offers a novel topical approach that may promote tissue repair and accelerate healing in refractory cases.

**Case presentation:**

This report describes four patients with persistent DFUs that responded poorly to conventional therapies and faced either prior amputation or an imminent risk of amputation. Ulcer sizes ranged from 3.0 cm × 3.0 cm to 7.0 cm × 5.0 cm, with comorbidities including hypertension and cardiovascular disease. In addition to routine wound care, patients received topical HFSJO treatment every other day. Within 30–180 days, all wounds showed substantial granulation, epithelialization, and eventual closure. No clinically significant abnormalities in the liver or renal function tests or routine hematological parameters were observed during treatment or follow-up.

**Conclusion:**

Topical application of HFSJO appears to be a safe and effective conservative strategy for the management of complex DFUs, particularly in patients unsuitable for surgery or unresponsive to standard care. Through its dual mechanism, i.e., targeted debridement and regenerative stimulation, it may help overcome healing stagnation. These cases offer a promising direction for integrative wound care and warrant further validation in controlled clinical trials.

## 1 Introduction

Diabetic foot (DF) is one of the most disabling and economically burdensome chronic complications of diabetes, driven primarily by peripheral neuropathy, lower limb ischemia, and immune dysfunction (1). Among its clinical manifestations, diabetic foot ulcers (DFUs) are the most prevalent, which are characterized by slow healing, high recurrence rates, and a significant risk of infection, gangrene, and eventual lower limb amputation. Epidemiological studies estimate that approximately 19%–34% of individuals with diabetes will develop DFUs during their lifetime, with up to 20% ultimately requiring amputation (2). Moreover, the 1- and 5-year mortality rates following DFU onset reach 10% and 30%, respectively, while post-amputation 5-year mortality further escalates to nearly 50% (3). Alarmingly, even healed DFUs carry a high recurrence risk—reported at 40% within 1 year and up to 65% within 5 years—leading clinicians to increasingly consider DFUs as a “remission-prone” rather than a “curable” condition (4).

Although standard therapeutic strategies, encompassing debridement, infection control, glycemic management, offloading, revascularization, and multidisciplinary approaches, are widely applied, several challenges remain in real-world clinical settings (5). For instance, some patients exhibit poor wound healing due to inadequate local perfusion and impaired immune response, resulting in chronic, low-inflammatory ulcers with deficient granulation and limited responsiveness to conventional dressings or antibiotics (6, 7). Others may suffer from recurrent infections and progressive necrosis due to inadequate antibiotic penetration under compromised microcirculation, ultimately necessitating multiple surgical debridements or amputations, which further exacerbate tissue damage and hinder wound closure (8, 9). Therefore, strategies aimed at reactivating local regenerative mechanisms and minimizing reliance on invasive interventions are urgently needed.

In recent years, traditional Chinese external therapies have attracted growing interest for their potential to modulate inflammation and promote tissue regeneration in chronic non-healing wounds. As early as in classical texts such as *Waike Zhengzong* and *Liu Juanzi Gui Yifang*, the concept of “Qu Fu Sheng Ji” (removing necrosis and promoting tissue growth) has been proposed for the treatment of ulcerative lesions. The Huafu Shengji ointment (HFSJO) was developed in accordance with this principle, which is composed primarily of mineral-based agents including realgar (As_4_S_4_), cinnabar (HgS), and alum [KAl(SO_4_)_2_ 12H_2_O], and is traditionally used for detoxification, necrotic tissue clearance, and stimulation of granulation. Although concerns over the potential toxicity of the heavy metal components have led to its marginalization in modern clinical practice, recent studies suggest that, when used topically in controlled dosages, such formulations do not exhibit significant systemic toxicity and may instead demonstrate immunoregulatory, debridement-promoting, and pro-granulation effects (10–12).

Based on these findings, the present study reports on four cases of refractory DFUs treated with standard care in combination with HFSJO. These cases were selected for detailed presentation as they had complete longitudinal clinical documentation and standardized photographic records, allowing clear illustration of wound progression. Case selection was based on data completeness rather than treatment response or clinical outcome. The aim was to explore the potential clinical value of this traditional formulation in enhancing wound debridement, promoting limb preservation, and reactivating ulcer healing in cases unresponsive to conventional therapy.

## 2 Patient information and clinical findings

### 2.1 Case 1

**Figure 1:**
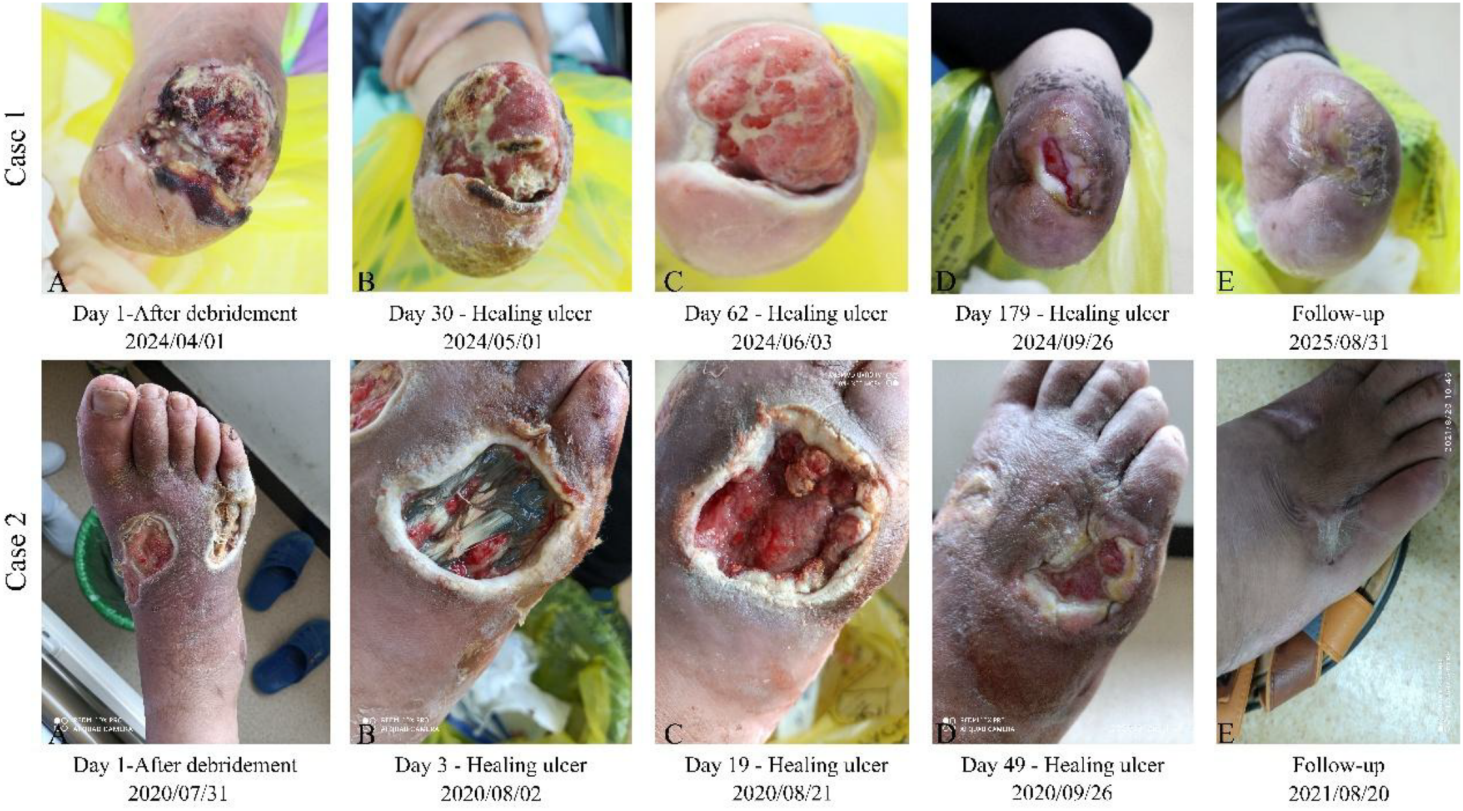
Changes in the healing of the ulcer in cases 1 and 2.

An 82-year-old woman with a >20-year history of diabetes mellitus presented with a 3-month history of a progressively worsening ulcer of the left foot, characterized by fever, progressive swelling, and purulent drainage. She had undergone multiple amputations of the left foot at Daxing District Hospital. However, the stump ulcer persisted with non-healing, prompting referral to our institution for conservative management. The patient was routinely taking metformin hydrochloride tablets orally (0.5 g, three times daily) and glipizide (2.5 mg, administered before meals). She reported unintentional weight loss with otherwise preserved nutritional status and intermittent palpitations and hand tremor. There was no other significant past medical history. Additional comorbidities included atherosclerosis, hepatic steatosis, reflux esophagitis, and chronic obstructive pulmonary disease. On local examination of the left foot, the amputation stump exhibited erythema, increased skin temperature, tenderness, and sanguineous oozing, without malodor (**Figure 1**). Imaging examination revealed stenosis of the dorsalis pedis artery and compromised blood flow in the patient (**Supplementary Figure 1**). According to the Wagner classification, the ulcer was graded as stage 4. Laboratory investigations showed fasting plasma glucose of 17.0 mmol/L and glycated hemoglobin (HbA1c) of 8.6%. The inflammatory and hematologic parameters were notable for C-reactive protein, 196.1 mg/L (elevated); neutrophils, 7.0 × 10^9^/L (elevated); white blood cells (WBCs), 9.8 × 10^9^/L (elevated); red blood cells (RBCs), 3.3 × 10^12^/L (decreased); and hemoglobin, 94.0 g/L (decreased). All other hematologic indices were within normal limits. Culture of wound exudate at 48 h demonstrated no growth of pathogenic organisms. Based on the International Working Group on the Diabetic Foot (IWGDF)/Infectious Diseases Society of America (IDSA) classification, the infection was classified as moderate in severity. The hepatic and renal function parameters are summarized in **Table 1**.

**Table 1:** Liver and kidney function indicators of the patients pre- and post-treatment.

Parameter	Normal range	Pre-treatment	Post-treatment
Case 1			
Total protein (g/L)	65–85	63.0	70.0
Albumin (g/L)	40–55	29.0	43.5
Albumin-to-globulin ratio	1.2–2.4	0.85	1.2
Cholinesterase (U/L)	5,000–12,000	3,361	6,854
Creatinine (μmol/L)	Female: 45–80	83	72
Urea (mmol/L)	3.2–7.1	12.3	6.8
Case 2			
Albumin (g/L)	40–55	34.9	44.9
Albumin-to-globulin ratio	1.2–2.4	1.02	1.31
Case 3			
Albumin (g/L)	40–55	24.0	43.4
Globulin (g/L)	20–40	41.1	36.5
Albumin-to-globulin ratio	1.2–2.4	0.58	1.19
Case 4			
Albumin (g/L)	40–55	28.5	42.5
Direct bilirubin (μmol/L)	0–6.8	8.2	3.4

All other hepatic and renal function parameters not listed above were within normal limits.

### 2.2 Case 2

A 37-year-old man with no history of hypertension presented with a dorsal right foot ulcer with exposed tendon, accompanied by exudation and swelling. At 1 year earlier, during fixation surgery for a right calcaneal fracture at the Eighth Medical Center of the PLA General Hospital, hyperglycemia was noted (exact values unavailable). He did not subsequently undergo systematic glucose monitoring. The patient had been receiving insulin therapy for glycemic control, consisting of subcutaneous insulin aspart 20 U three times daily and subcutaneous insulin glargine 15 U nightly at bedtime. He is a taxi driver working 10 h daily. Following divorce, he is the sole caregiver for his daughter. Given the substantial socioeconomic burden, he sought limb salvage (conservative) management at our institution to avoid amputation. On examination, the right foot was edematous with dusky discoloration. Two infected ulcers were present, measuring approximately 7 cm × 5 cm and 6 cm × 6 cm. The lateral lesion extended to the fascia, with exudate, malodor, and partial sloughing of necrotic tissue (**Figure 1**). Imaging studies revealed no apparent abnormalities in blood flow to the lower extremity (**Supplementary Figure 2**), whereas neurological examination demonstrated reduced tactile sensation in the foot. According to the Wagner classification, the ulcer was graded as stage 3. Past and family histories were otherwise unremarkable. The patient was well developed and well nourished. Blood pressure was 120/80 mmHg and heart rate was 80 bpm, regular, without murmurs. Laboratory evaluation showed HbA1c 9.9%, and complete blood count revealed no significant abnormalities. Culture of wound exudate at 48 h demonstrated no growth of pathogenic organisms. According to the IWGDF/IDSA classification, the infection was categorized as moderate. The hepatic and renal function indices are summarized in **Table 1**.

### 2.3 Case 3

**Figure 2:**
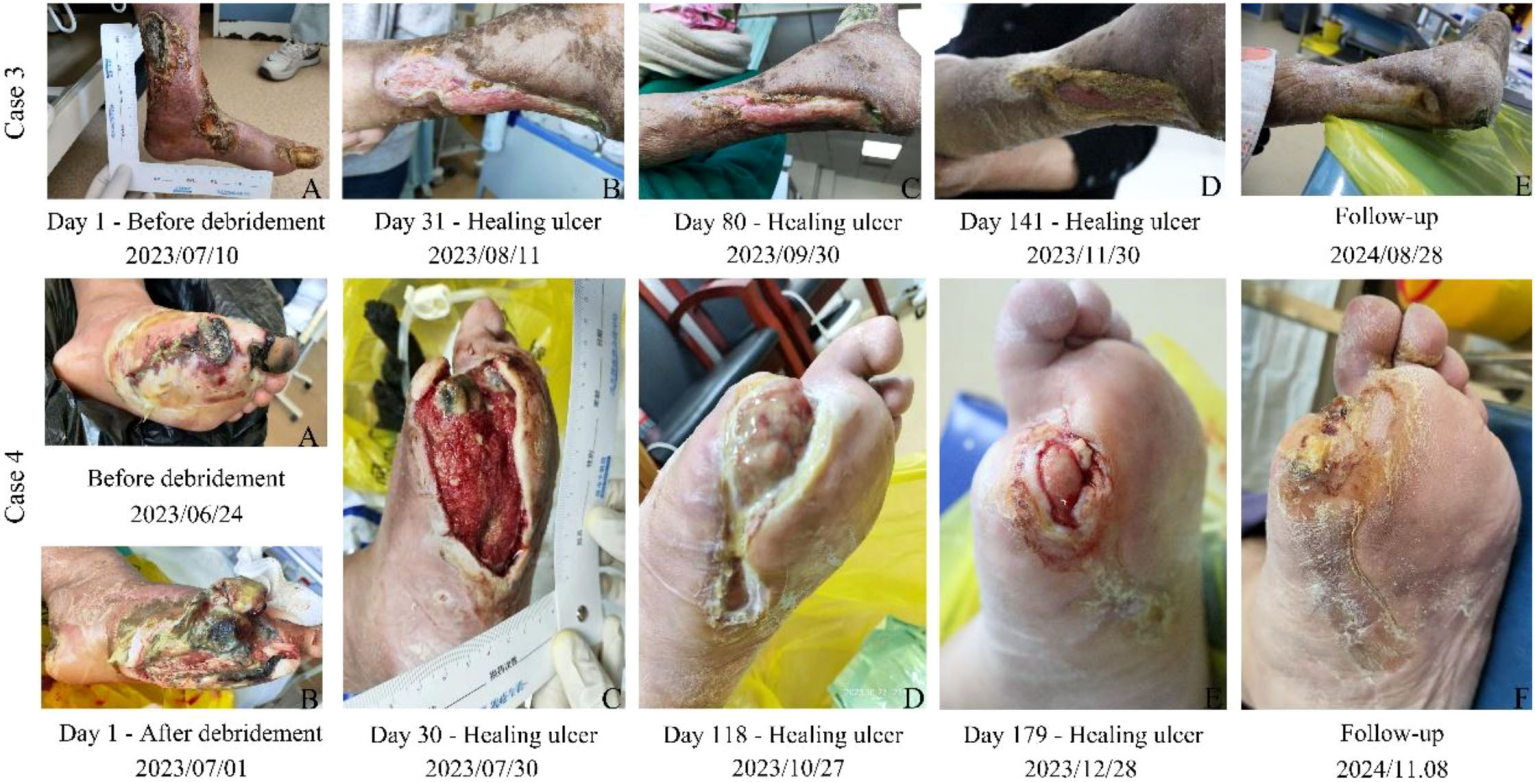
Changes in the healing of the ulcer in cases 3 and 4.

An 86-year-old woman with a >40-year history of type 2 diabetes mellitus and hypertension presented with a 6-month history of left foot edema accompanied by ulceration. Her family had performed unsupervised dressing changes at home (agents unspecified) without symptom relief; the condition progressively worsened. In view of extensive tissue necrosis but a strong preference for limb preservation, she sought limb salvage conservative management at our institution. The patient was routinely taking acarbose tablets orally (50 mg, taken with meals). She was thin with fair nutritional status and had no additional pertinent past medical history. Other diagnoses included status post-coronary artery bypass grafting, severe osteoporosis, grade 3 hypertension in the very high-risk category, and anemia. Foot examination revealed multiple ulcers of the left foot: three lesions measuring approximately 12 cm × 12 cm and multiple digital ulcers measuring approximately 3 cm × 2 cm. Wound depth extended to the muscle layer, with areas of exposed bone in the larger defects. The ipsilateral lower leg was erythematous and edematous with increased skin temperature. The patient reported no significant foot pain. The wounds exuded yellow purulent discharge (**Figure 2**). Imaging studies revealed severe occlusion of the anterior tibial artery, occlusion of the posterior tibial artery, and findings consistent with DF (**Supplementary Figure 3**). Bacterial culture identified *Proteus mirabilis*. Based on the IWGDF/IDSA classification, the infection was classified as moderate in severity. Neurologic assessment demonstrated diminished tactile sensation. According to the Wagner classification, the ulcer was graded as stage 4. Laboratory evaluation showed fasting blood glucose of 7 mmol/L and HbA1c of 6.3 mmol/L (as reported), blood pressure of 161/60 mmHg, reduced RBC count of 2.9 × 10^12^/L, a markedly decreased hemoglobin of 69.0 g/L, and a decreased mean corpuscular volume of 77.0 fl. All other hematologic indices were within normal limits. A standard cardiac enzyme panel and the coagulation studies were unremarkable. The hepatic and renal function indices are summarized in **Table 1**.

### 2.4 Case 4

A 42-year-old woman with a >20-year history of diabetes mellitus presented with a 2-week history of ulceration of the left foot that had rapidly worsened over the preceding 3 days, characterized by black discoloration, swelling, and a foul odor, prompting urgent evaluation at our institution. The patient had been receiving subcutaneous injections of biosynthetic human insulin isophane before breakfast and dinner, at doses of 20 U before breakfast and 20 U before dinner. She had an average body habitus with good nutritional status and no other notable past medical history. Family history was positive for diabetes in both parents. Additional diagnoses included atherosclerotic heart disease, hyperlipidemia, and bronchial asthma. Local examination revealed a 2-cm × 2-cm necrotic (black) lesion over the first metatarsophalangeal joint, a 5-cm × 5-cm purulent ulcer on the dorsal aspect of the left foot, and a 3-cm × 6.5-cm ulcer on the plantar surface. These findings were accompanied by lower limb edema, increased local skin temperature, and malodor (**Figure 2**). Imaging studies revealed perfusion defects with intermittent blood flow signals, as well as soft tissue infection, soft tissue necrosis, and osteonecrosis of the foot (**Supplementary Figure 4**). Wound exudate culture grew *Staphylococcus aureus*. Based on the IWGDF/IDSA classification, the infection was classified as moderate in severity. Neurologic assessment demonstrated a diminished tactile sensation. According to the Wagner classification, the ulcer was graded as stage 4. Laboratory studies showed an elevated HbA1c of 10.6%, procalcitonin of 1.0 ng/ml (elevated), WBC count of 23.3 × 10^9^/L with 84.9% neutrophils, and hemoglobin of 91.0 g/L. All other hematologic parameters were within normal limits. The hepatic and renal function indices are summarized in **Table 1**.

## 3 Diagnostic assessment

All DFUs were staged using the Wagner system: cases 1, 3, and 4 met the criteria for grade 4, while case 2 met the criteria for grade 3. At bedside evaluation, each lesion was profiled for ulcer size, morphology, margin characteristics, base, and depth. Routine blood tests were obtained concurrently.

## 4 Therapeutic intervention

Treatment was individualized according to ulcer severity (Wagner grade) and patient baseline status (e.g., diabetes classification and infection burden). A multimodal regimen integrating guideline-directed standard care with traditional Chinese medicine (TCM)-based external therapies (Huafu Shengji Ointment) was employed (**Table 2**) and delivered within an internal–external co-treatment framework to enhance wound healing.

**Table 2:** Treatment plan for a patient with diabetic foot ulcer.

System of medicine	Plan of treatment
Case 1	
Modern therapies	Antibiotics: norvancomycin hydrochloride for injection combined with imipenem and cilastatin sodium for injection: 1 g, administered every 12 hours
	Insulin therapy: insulin degludec and aspart 28 U subcutaneously twice daily
	Amputation of the left foot under local anesthesia
TCM external therapies	Local debridement: iodophor disinfection, saline cleaning, removal of necrotic tissue, and wound exposure
	Acupuncture: bilateral LI4 (Hegu), SP6 (Sanyinjiao), ST36 (Zusanli)
	Huafu Shengji ointment: Applied in a 1-mm layer under sterile gauze dressing, changed every other day
Medication during the follow-up period	Antibiotics: levofloxacin tablets (0.5 g, once daily)
	Insulin and hypoglycemic drug therapy: glimepiride tablets (2.0 mg, once daily); acarbose tablets (50 mg, orally before meals, three times daily)
	Huafu Shengji ointment: Applied in a 1-mm layer under sterile gauze dressing, changed every other day
Case 2	
Modern therapies	Antibiotics: levofloxacin hydrochloride and sodium chloride injection: 0.4 g via intravenous infusion once daily
	Insulin therapy: insulin degludec and aspart 16 U via continuous subcutaneous infusion by insulin pump three times daily, combined with oral metformin (0.5 g, three times daily)
TCM external therapies	Local debridement: iodophor disinfection, saline cleaning, removal of necrotic tissue, and wound exposure
	Acupuncture: bilateral LI4 (Hegu), SP6 (Sanyinjiao), ST36 (Zusanli)
	Huafu Shengji ointment: Applied in a 1-mm layer under sterile gauze dressing, changed every other day
Medication during the follow-up period	Antibiotics: levofloxacin hydrochloride tablets (0.5 g, once daily)
	Insulin and hypoglycemic drug therapy: insulin aspart injection (8 U, three times daily, inject immediately before meals); insulin detemir injection (14 U, once daily at bedtime); metformin hydrochloride tablets (0.5 g, three times daily, take with meals)
	Huafu Shengji ointment: Applied in a 1-mm layer under sterile gauze dressing, changed every other day
Case 3	
Modern therapies	Antibiotics: piperacillin and tazobactam sodium 2.5 g every 12 h
	Insulin therapy: acarbose 50 mg three times daily; linagliptin 5 mg twice daily
TCM external therapies	Local debridement: iodophor disinfection, saline cleaning, removal of necrotic tissue, and wound exposure
	Acupuncture: bilateral LI4 (Hegu), SP6 (Sanyinjiao), ST36 (Zusanli)
	Huafu Shengji ointment: Applied in a 1-mm layer under sterile gauze dressing, changed every other day
Medication during the follow-up period	Antibiotics: –
	Insulin and hypoglycemic drug therapy: empagliflozin tablets (10 mg, once daily); acarbose tablets (50 mg, orally before meals, three times daily)
	Huafu Shengji ointment: Applied in a 1-mm layer under sterile gauze dressing, changed every other day
Case 4	
Modern therapies	Antibiotics: imipenem for injection (1 g every 8 h) combined with vancomycin for injection (0.8 g twice daily)
	Insulin therapy: continuous glucose management via insulin pump (insulin degludec and aspart 28 U)
TCM external therapies	Local debridement: dry gangrene of the left hallux with spontaneous demarcation and auto-amputation; remaining areas treated with iodophor disinfection, saline irrigation, debridement, and wound exposure
	Acupuncture: bilateral LI4 (Hegu), SP6 (Sanyinjiao), ST36 (Zusanli)
	Huafu Shengji ointment: Applied in a 1-mm layer under sterile gauze dressing, changed every other day
Medication during the follow-up period	Antibiotics: –
	Insulin and hypoglycemic drug therapy: insulin degludec and aspart injection (subcutaneous injection, 28 U in the morning, 26 U in the evening); metformin hydrochloride tablets (0.5 g, three times daily)
	Huafu Shengji ointment: Applied in a 1-mm layer under sterile gauze dressing, changed every other day

*TCM*, traditional Chinese medicine.

## 5 Follow-up and outcome

Management was individualized to each patient’s clinical presentation and disease severity (Wagner grade). During the treatment phase, infection control and hyperglycemia were addressed with conventional Western medical therapy, while a TCM topical approach, HFSJO, applied for its debriding and granulation-promoting effects, was used to maximize limb salvage and expedite wound closure, thereby improving quality of life (**Table 2**). Across the four cases, DFUs were effectively controlled as assessed longitudinally using the Bates–Jensen wound assessment tool (BWAT) (**Table 3**). BWAT scoring was performed by the attending physician based on the wound data collected by trained nursing staff according to standardized procedures. All cases ultimately achieved complete closure (**Figures 1**, **2**). Laboratory indices—including albumin, globulin, bilirubin, urea, and serum creatinine—normalized after therapy (**Table 1**). At the 1-year follow-up, all patients showed sustained recovery with improved quality of life and reduced daily burden, and they maintained glycemic control on the same therapeutic regimen in routine practice.

**Table 3:** Assessment of various ulcer parameters with the Bates–Jensen wound assessment tool.

Day	Size	Depth	Edges	Undermining	Necrotic tissue type	Necrotic tissue amount	Exudate type	Exudate amount	Skin color surrounding wound	Peripheral tissue edema	Peripheral tissue induration	Granulation tissue	Epithelialization	Total score
Case 1														
1–30	3	3	4	3	3	3	2	4	3	2	5	4	5	44
30–60	3	2	3	3	2	2	2	3	3	2	4	3	4	36
60–90	2	2	2	2	1	1	1	2	2	1	2	2	2	22
90–180	1	1	1	1	1	1	1	1	1	1	1	1	1	13
Case 2														
1–15	3	3	3	2	3	3	5	4	4	4	3	4	4	45
15–30	3	3	3	2	2	2	2	3	4	2	3	3	3	35
30–60	2	2	2	1	1	1	1	2	2	1	2	2	2	21
60–120	1	1	1	1	1	1	1	1	1	1	1	1	1	13
Case 3														
1–30	5	4	3	2	4	4	4	4	4	5	5	5	5	54
30–60	4	2	3	2	3	3	3	3	3	4	4	3	3	38
60–90	3	2	2	1	2	2	1	2	1	2	3	2	2	25
90–160	2	1	1	1	1	1	1	1	1	1	2	1	1	15
Case 4														
1–30	4	5	4	4	5	5	5	5	3	5	4	5	4	58
30–60	4	5	3	3	3	3	3	4	2	3	3	3	3	42
60–120	2	3	2	2	2	2	1	3	1	2	2	2	2	26
120–180	1	1	1	1	1	1	1	1	1	1	1	1	1	13

## 6 Discussion

This case series describes four cases of DFUs in which wound healing and limb preservation were achieved under multimodal standard-of-care management with the addition of HFSJO as a topical adjunct. Improvements were observed in wound-bed appearance, including necrotic tissue reduction and granulation formation, followed by progressive epithelialization and closure. Collectively, these clinical courses suggest that HFSJO may be a feasible treatment option in select refractory DFUs when conventional local wound care yields limited progress.

The unique value of HFSJO in DFU treatment lies in its dual “debridement” mechanism. Firstly, in ulcers with extensive necrosis and a high risk of amputation, HFSJO appears to facilitate selective separation of devitalized tissue while preserving adjacent viable structures, consistent with a self-limiting debridement-like effect. This function may reduce the need for repeated surgical intervention and minimize trauma to healthy tissues, thereby improving limb salvage outcomes. Secondly, in ulcers that had entered a “stagnant” phase, characterized clinically by minimal granulation, poor responsiveness to conventional dressings, and persistent non-healing, the local pharmacologic stimulation from HFSJO appears to reactivate the wound healing dynamics. Clinically, this is evidenced by a transition from a pale, inert wound bed to one with hyperemic granulation, indicating a shift from a chronic low-inflammatory state to a controlled pro-inflammatory, regenerative microenvironment. This dual mechanism addresses two critical bottlenecks in chronic wound care: effective selective debridement and reactivation of stalled wounds.

From a practical clinical perspective, HFSJO is generally considered when routine local management failed to produce sustained improvement, particularly in wounds that remained refractory despite standard care or repeatedly redeveloped slough after debridement, resulting in prolonged stagnation. In this setting, HFSJO served not as a replacement for systemic management but as a local wound treatment introduced when conventional measures alone were insufficient to restore healing progression. Its role is to support a staged transition in the wound course: firstly by promoting clearer demarcation and gradual separation of nonviable tissue and then by facilitating granulation and progressive epithelial repair once the wound bed becomes more viable. The clinical objective was to help long-standing non-healing ulcers reenter an active healing process and ultimately achieve complete wound healing, defined in this study as full epithelialization without exudation. Compared with previously published case-based reports of conservative DFU management (13), the treatment course in our series appeared relatively longer, which should be interpreted in light of the greater baseline complexity of the wounds and the broader therapeutic goals in our cases.

The relatively prolonged treatment duration observed in some cases should therefore not be viewed simply as delayed response. The ulcers in this series had typically remained unhealed for several months and, in some cases, for more than 1 year before HFSJO was introduced. In addition, a number of patients presented with marked swelling, extensive tissue compromise, and impending amputation, but strongly desired limb preservation. In such cases, treatment necessarily began with a prolonged preparatory phase focused on progressive separation of the fully devitalized tissue while preserving as much residual viable foot structure and function as possible. Only after this stage could granulation and epithelialization gradually proceed. Accordingly, the longer treatment course reflects the staged management of highly complex refractory DFUs, particularly when the priority is to maximize tissue preservation rather than proceed directly to ablative surgery. This also helps explain why the healing timeline in our series differs from that reported in other case reports.

Although all patients have received multimodal systemic management (e.g., antibiotics and glycemic control) to address infection burden and metabolic derangements, the only topical intervention applied directly to the wound bed has been HFSJO. Notably, wound improvement has been observed after the initiation of HFSJO following a period of limited response to prior supportive measures. In summary, this preliminary study highlights the clinical potential of HFSJO as a conservative therapeutic option for complex DFUs, especially in patients who are unsuitable for surgical intervention, prefer noninvasive strategies, fail to respond to conventional treatments, or seek maximal preservation of residual foot function in a limb salvage setting.

Safety considerations are particularly relevant as HFSJO has metal-containing components. In this series, routine hepatic and renal function indices and the hematological parameters did not show clinically meaningful deterioration from baseline during treatment and follow-up, and no obvious adverse events were identified on routine clinical monitoring, including local dermatologic reactions or neurological symptoms assessed in standard practice. Nevertheless, these observations are limited to the monitored clinical and laboratory parameters and do not substitute for direct quantification of systemic exposure (e.g., blood or urine arsenic/mercury levels) or dedicated assessments of cumulative heavy metal toxicity. Accordingly, the safety interpretations in this report are restricted to the reported findings, and future studies should incorporate standardized adverse event reporting, longer follow-up, and direct heavy metal exposure assessments. In our ongoing work, we are prospectively collecting samples for external quantitative testing of arsenic and mercury, and safety monitoring will be further strengthened in our concurrently running controlled studies.

Despite these limitations, the present cases indicate that HFSJO may warrant further investigation as a minimally invasive topical adjunct within an integrated wound care framework. Future large-scale, multicenter, controlled clinical trials with standardized outcome measures, such as wound area reduction over time, time to complete epithelialization, infection control endpoints, and limb salvage, are needed to validate efficacy, clarify mechanisms, and define appropriate indications and safety monitoring requirements. Within the broader context of multidisciplinary and individualized wound management, HFSJO, representing a classical modality in traditional Chinese topical therapy, may serve as a promising treatment in the management of chronic non-healing wounds.

## Data Availability

The raw data supporting the conclusions of this article will be made available by the authors, without undue reservation.
