# Supplementary material for: Curative outcomes with metal-containing TCM in diabetic foot ulcers unresponsive to standard therapy: a case series
Source: Front Endocrinol (Lausanne). 2026 May 8;17:1736055. doi: 10.3389/fendo.2026.1736055 (PMC13193884; doi:10.3389/fendo.2026.1736055)
Supplement: Supplementary file 1 [file DataSheet1.docx]

Supplementary 1


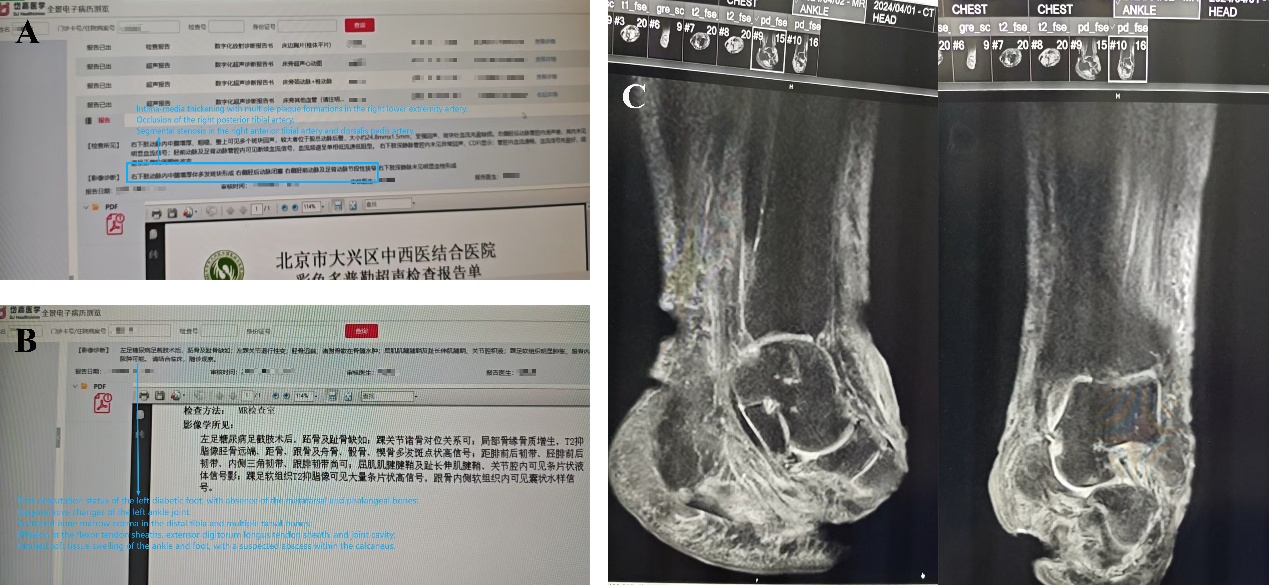


Figure 1 Imaging reports and images of Case 1.​ (A) Spectral Doppler ultrasound report, showing stenosis of the dorsalis pedis artery with restricted blood flow. (B) Foot MRI report, demonstrating absence of the metatarsal and phalangeal bones following amputation for diabetic foot, with a cystic fluid signal noted in the soft tissues adjacent to the calcaneus. (C) Foot MRI scan.

Supplementary 2


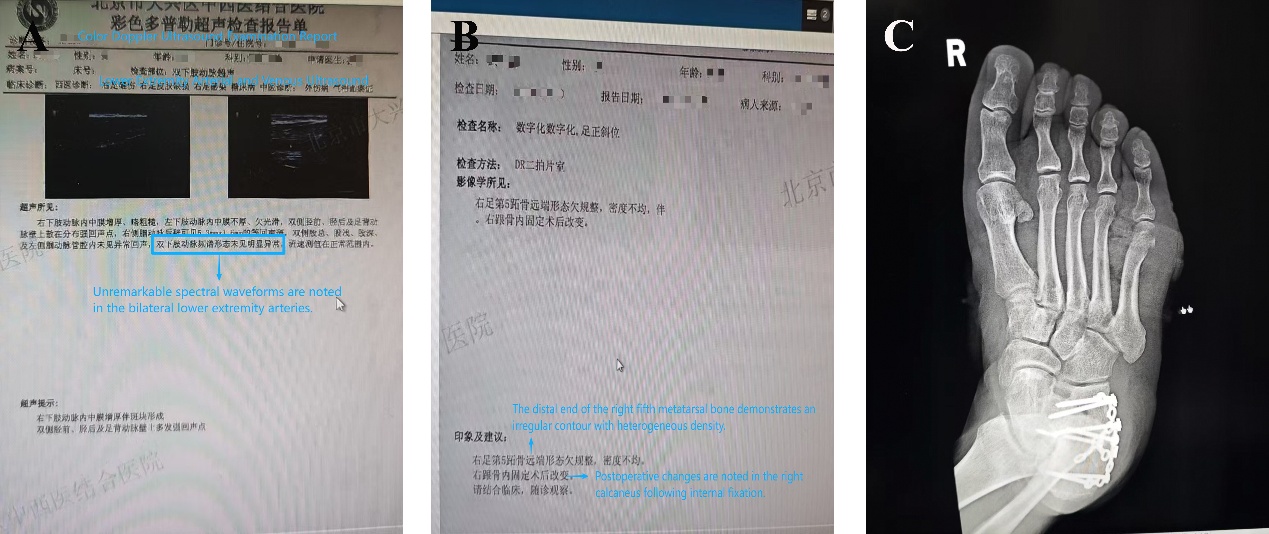


Figure 2 Imaging reports and images of Case 2.​ (A) Color Doppler ultrasound report, showing unremarkable blood flow in the lower extremity. (B) Foot DR (digital radiography) report. (C) Foot DR image.

Supplementary 3


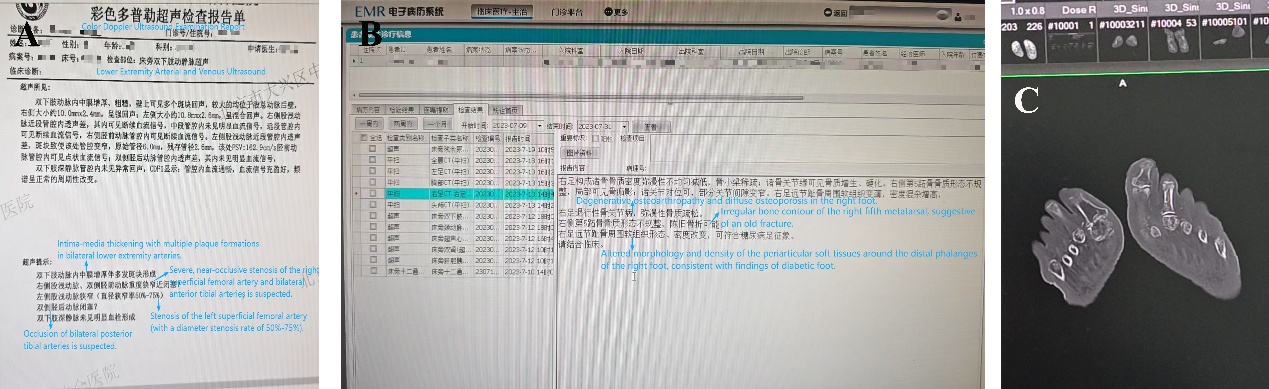


Figure 3 Imaging reports and images of Case 3. (A) Color Doppler ultrasound report, showing severe occlusion of the anterior tibial artery and occlusion of the posterior tibial artery. (B) Foot CT report, demonstrating findings of diabetic foot. (C) Foot image.

Supplementary 4


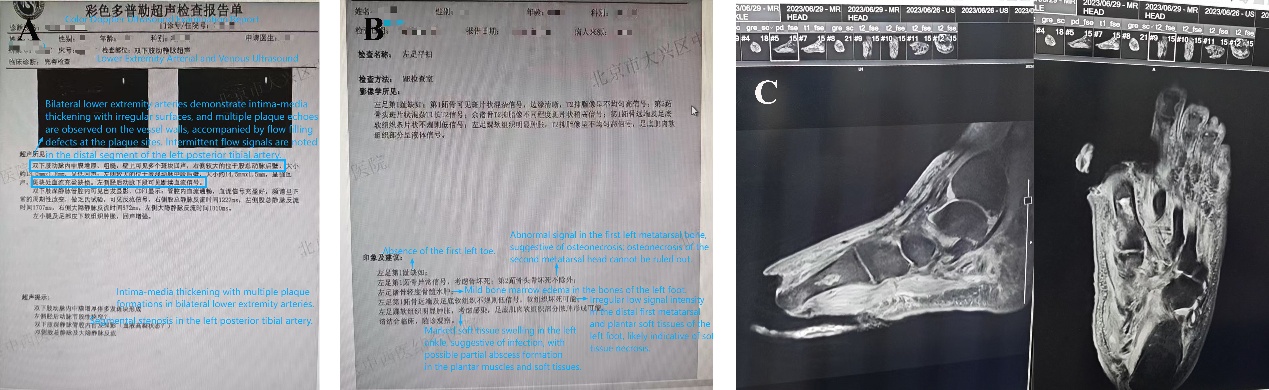


Figure 4 Imaging reports and images of Case 4. (A) Color Doppler ultrasound report, showing flow filling defects with intermittent flow signals observed. (B) Foot MRI report, demonstrating findings of soft tissue infection, soft tissue necrosis, and osteonecrosis in the foot. (C) Foot image.
